# When Argonaute takes out the ribonuclease sword

**DOI:** 10.1016/j.jbc.2023.105499

**Published:** 2023-11-27

**Authors:** Kotaro Nakanishi

**Affiliations:** 1 Department of Chemistry and Biochemistry, The Ohio State University, Columbus, Ohio, USA; 2 Center for RNA Biology, The Ohio State University, Columbus, Ohio, USA

**Keywords:** Argonaute, microRNAs, RISC, endonuclease

## Abstract

Argonaute (AGO) proteins in all three domains of life form ribonucleoprotein or deoxyribonucleoprotein complexes by loading a guide RNA or DNA, respectively. Since all AGOs retain a PIWI domain that takes an RNase H fold, the ancestor was likely an endoribonuclease (*i.e.*, a slicer). In animals, most miRNA-mediated gene silencing occurs slicer independently. However, the slicer activity of AGO is indispensable in specific events, such as development and differentiation, which are critical for vertebrates and thus cannot be replaced by the slicer-independent regulation. This review highlights the distinctions in catalytic activation mechanisms among slicing-competent AGOs, shedding light on the roles of two metal ions in target recognition and cleavage. The precision of the target specificity by the RNA-induced silencing complexes is reevaluated and redefined. The possible coevolutionary relationship between slicer-independent gene regulation and AGO-binding protein, GW182, is also explored. These discussions reveal that numerous captivating questions remain unanswered regarding the timing and manner in which AGOs employ their slicing activity.

More than 2000 miRNAs have been reported in humans (1). The genes of miRNAs are transcribed as stem–loop structured RNAs, called primary miRNA, and undergo processing by microprocessor, which is a complex of RNase III enzyme Drosha and its binding protein DGCR8, in the nucleus (Fig. 1*A*) (2). The products, precursor miRNAs, are transported to the cytoplasm. Their loop is cropped by Dicer, a molecular ruler that generates size-specific miRNA duplexes (3). The resultant miRNA duplexes have a length of about 22-nts with 2-nt 3′ overhangs at both ends, the feature that licenses loading into Argonaute (AGO) proteins. One strand of the duplex, called the passenger strand, is ejected while the remaining guide strand and AGO form the ribonucleoprotein complexes called RNA-induced silencing complexes (RISCs) (Fig. 1*B*) (4). The RISC assembly establishes the standardized guide segmentation, which is composed of the seed (guide nucleotide positions 2–8: g2–g8), central (g9–g12), 3′ supplementary (g13–g16), and tail regions (g17–3′ end) (Fig. 1, *B* and *C*). Human Argonaute2 (*Hs*AGO2) cleaves a complementary target RNA between target positions 10 and 11 (t10 and t11), which form Watson–Crick base pairs with g10 and g11, respectively (Fig. 1*C*) (5, 6). In contrast, *Hs*AGO3 is catalytically activated by ∼14-nt specific short guide RNAs (see below) (7, 8). RISCs facilitate their silencing activity by interacting with GW182, an AGO-binding protein that recruits the CCR–NOT4 complex to shorten poly(A) of the RISC-bound mRNAs. The biogenesis and degradation of miRNAs and siRNAs, as well as the roles of RISC in posttranscriptional gene silencing and translational repression, have been studied for the last two decades (and summarized elsewhere (2, 4, 9, 10, 11, 12, 13, 14)). The current review will mainly focus on the endoribonuclease activity of AGOs.

**Figure 1:**
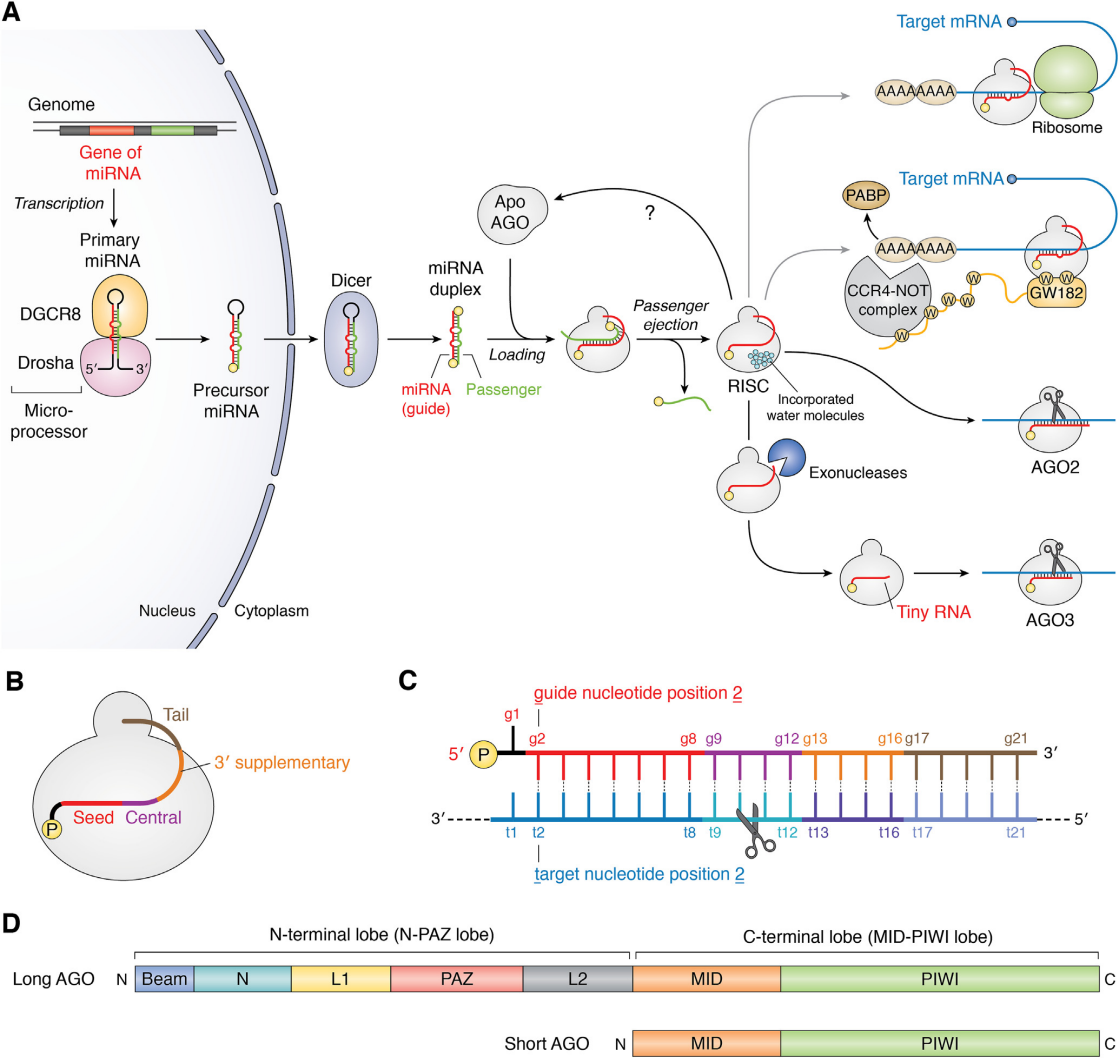
**MicroRNA biogenesis and their roles**. *A*, lives of miRNAs. After processing by Dicer, the miRNA duplex is loaded into Argonaute (AGO) proteins. During RISC assembly, AGOs take in water molecules to affix the domains (35). Slicer-dependent and slicer-independent processes are indicated as *black* and *gray lines*, respectively. Specific 3′→5′ exonucleases trim AGO-associated miRNAs to tiny RNAs (77). Only AGO2 and AGO3 become slicers (8), though physiological target RNAs cleaved by AGO3-RISC remain unknown. *Yellow spheres* represent a 5′ monophosphate group of guide RNAs. *B*, schematic of mature RISC composed of an AGO (*white*) and a mature miRNA. The standardized guide segmentation is shown with color codes: seed (*red*), central (*magenta*), 3′ supplemental (*orange*), and tail (*wheat*). *C*, nomenclature of guide and target nucleotide positions. The color codes of the guide segmentation are the same as (*B*). *D*, domain architectures of long AGO and short AGO. AGO, Argonaute; RISC, RNA-induced silencing complex.

## The bilobed AGO structure is essential for guide-dependent endonuclease activity

All eukaryotic AGOs are long AGOs composed of a single polypeptide encompassing the six domains (*i.e.*, N, L1, PAZ, L2, MID, and PIWI), which folds into a bilobed RISC architecture with the aid of a guide RNA (Fig. 1*D* top) (4, 13). On the other hand, prokaryotes have another type of AGO composed of a MID and PIWI domain, shaping a unilobed structure (Fig. 1*D* bottom) (15). This short AGO has an incomplete or absent catalytic DEDH/D tetrad and works as a bacteriophage sensor (16). In the AGO history, two key events led guide-dependent endonuclease AGOs (slicer-competent AGOs) to become the standard machinery across the three domains of life (17). First, an RNase H-like PIWI domain (18) was fused with a Rossmann fold–like MID domain (19). The resultant short AGOs could incorporate a single-stranded polynucleotide, forming a ribonucleoprotein or deoxyribonucleoprotein complex. The bound single strand serves as a guide to find complementary targets. In addition, short AGOs have recognition determinants to determine the target nucleotide positions from the 5′ end of the guide locked at the MID domain (20, 21). These features differentiate short AGOs from RNase H because RNase H can bind only to preformed DNA-RNA hetero duplexes and does not have a system to determine and cleave a specific position on the RNA strand (22). The second event was that an ancestor of long AGOs gained the intervening proteinaceous channel that works as the extensible platform for target recognition and cleavage. The acquisition of the bilobed structure enables the long AGOs to recognize mismatches and thus establish whether the bound target should be cleaved or not. Using a slicer-competent long AGO from yeast as a template, we made a fragment of an isolated C-terminal lobe, mimicking a short AGO (23). This construct retained decent slicer activity but cleaved partially complementary RNAs. Therefore, it seems reasonable that most short AGOs lost their catalytic residues during evolution (17), perhaps to eliminate a promiscuous and harmful cleavage of cellular nucleic acids. The result also demonstrates that the nucleic acid–binding channel between the two lobes is essential to avoid off-target cleavage by recognizing a mismatch between the guide and target strands (23). Prokaryotic and eukaryotic long AGOs have fine-tuned their nucleic acid–binding channel and developed various mechanisms for target recognition and cleavage specificity.

## The composite target-binding channel defines the target specificity

**Figure 2:**
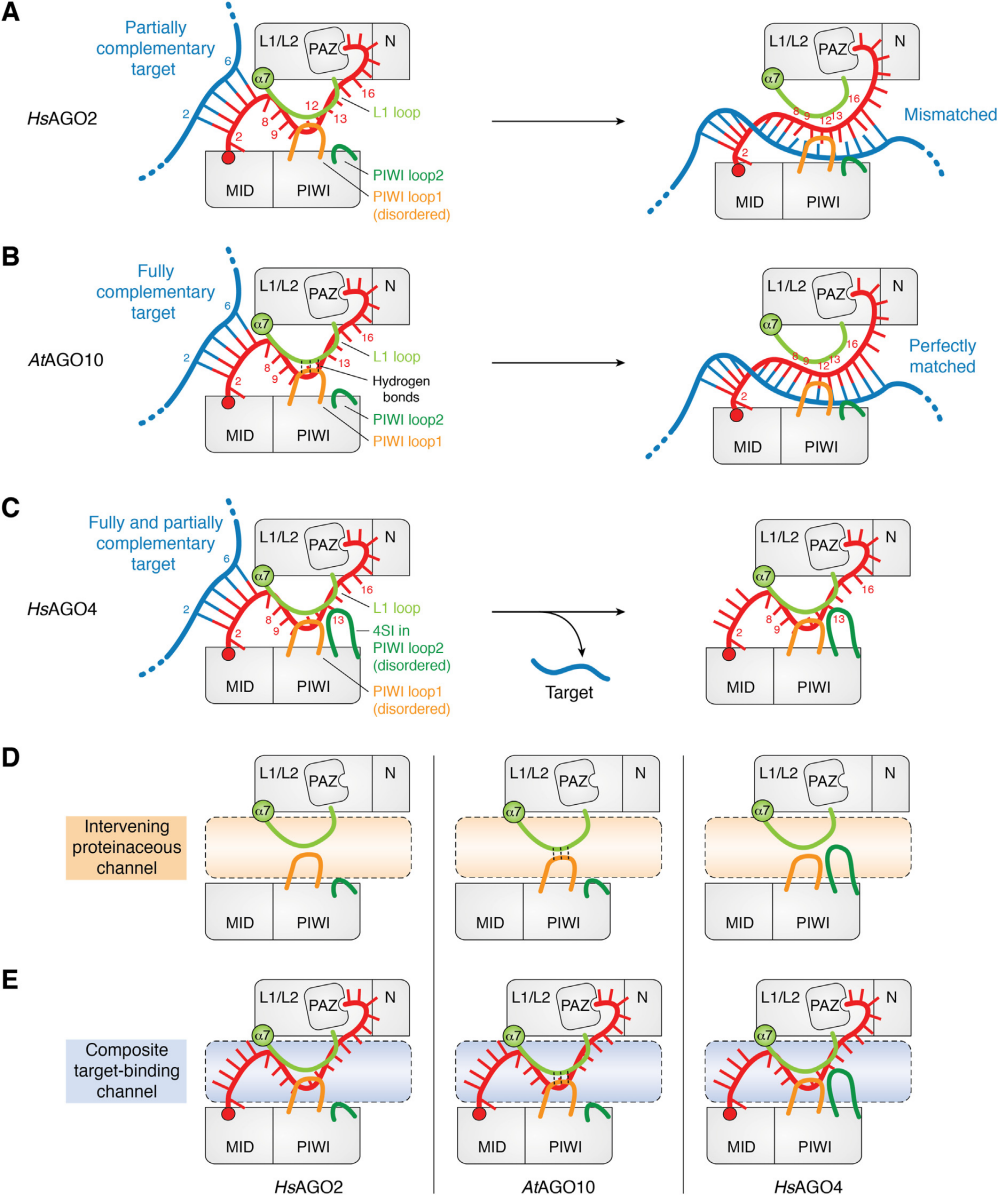
**The AGO channel defines the target specificity of the RISC.***A*–*C*, schematics of the different methods of target recognition by *Hs*AGO2 (*A*) (36, 61), *At*AGO10 (*B*) (33), and *Hs*AGO4 (*C*) (35). *Hs*AGO2 has a flexible PIWI loop1, allowing partially complementary targets into the nucleic acid–binding channel (*i.e.*, the composite target-binding channel). *At*AGO10 accepts only fully complementary targets because the PIWI loop1 and the L1 loop lower target accessibility to the channel. *Hs*AGO4 does not bind to target RNAs tightly because the 4SI and the L1 loop cover the channel. *D*, differences in the intervening proteinaceous channel (*orange-shaded area*) among *Hs*AGO2, *At*AGO10, and *Hs*AGO4 due to their unique loops. The intervening proteinaceous channel is formed by the N-PAZ lobe and the MID-PIWI lobe in the context of the bilobed RISC architecture but not in the context of apo-AGO. *E*, differences in the composite target-binding channel (*blue-shaded area*) among *Hs*AGO2, *At*AGO10, and *Hs*AGO4 are attributed to the differences in their intervening proteinaceous channels (*D*). All AGOs load the same guide RNA (*red*). The PIWI loop1 of *Hs*AGO2 (*A*) and *Hs*AGO4 (*C*) was disordered in their crystal structures. 4SI, AGO4-specific insertion; AGO, Argonaute; *At*AGO10, *Arabidopsis thaliana* AGO10; HsAGO, human Argonaute; RISC, RNA-induced silencing complex.

The algorithms to predict the binding site of guide RNAs search for complementary sequences on possible target RNAs (24, 25, 26, 27, 28, 29). This strategy was developed based on the consensus that the AGO-loaded guide recruits the RISC to partially or fully complementary targets (30, 31, 32). Metazoan miRNAs primarily use their guide nucleotide positions 2 to 7 or 2 to 8 (g2–g7 or g2–g8), called the seed region, to find target RNAs (12). The method of target recognition allows miRNAs to bind to mRNAs whose sequence is less complementary to the central (g9–g12) and 3′ supplementary regions (g13–g16) (Fig. 2*A*). This targeting enables mammalian miRNAs to interact with hundreds of different mRNAs (12, 32). In contrast, plant miRNAs require extensive complementarity through the seed, central, and part of 3′ supplementary regions (Fig. 2*B*) (33). Recent structural and functional studies on *Arabidopsis thaliana* AGO10 (*At*AGO10) visualized the bridging of the nucleic acid–binding channel by the L1 loop and PIWI loop1, which stick out from the L1 and PIWI domains, respectively (Fig. 2*B*) (33, 34). Since the bridged loops limit the access of target RNAs to the seed, *At*AGO10-RISC releases target RNAs unless the target is paired with the central and 3′ supplemental regions. This study provided the structural basis to understand the mechanism conferring high target specificity.

*Hs*AGO4 extends the PIWI loop2 with an AGO4-specific insertion (4SI) (Fig. 2*C*) (35). Unlike *At*AGO10, neither the PIWI loop1 nor loop2 forms hydrogen bonds with the L1 loop, but these three loops create a composite lid over the nucleic acid–binding channel. Therefore, the 4SI seems to reduce the access of target RNAs to the central and 3′ supplementary regions (Fig. 2*C*) rather than enhancing the extensive pairing with the target as seen in *At*AGO10. Indeed, the removal of the 4SI enabled the *Hs*AGO4 mutant to bind about 1.7-fold more target RNAs (35). Comparing these AGOs helps understand their distinct target recognition mechanisms attributed to the differences in their PIWI loops. All long AGOs fold into a typical bilobed architecture (4, 13), but their intervening proteinaceous channels have different shapes (Fig. 2*D*). Therefore, when considering the target specificity of a miRNA, we need to take into account the composite target-binding channel, which is the combination of the intervening proteinaceous channel and the loaded guide RNA (Fig. 2*E*). Supporting this idea, the crystal structure revealed that, unlike *At*AGO10, the central region of *Hs*AGO2-associated guide is not base paired even with the fully complementary target (36) because their proteinaceous channels are different. How HsAGO2 pairs the central region with a target to promote cleavage remains unclear. Presumably, variations in the intervening proteinaceous channel between AGOs also impact their preference for guide RNAs during RISC assembly. This notion could explain the differences in target specificity and guide selection across paralogs and orthologs (8, 37, 38, 39, 40, 41) and potential redundancy among human AGOs (42). Further *in vivo* studies are required to determine the suite of targets preferentially recognized by a specific AGO paralog.

## Recognition of the 5′ end nucleotide of guide RNA

A previous structural and functional study using the isolated *Hs*AGO2 MID domain with nucleoside monophosphate identified the nucleotide-specificity loop, which has a high affinity for adenine and uracil over cytosine and guanine (43). This study showed that the 5′ nucleotide preference resides in this loop. Likewise, *A. thaliana* AGO4 and AGO6 are known to load 24-nt siRNAs with a 5′ adenine (44). A recent study unveiled that *At*AGO4 has a moderate preference for a 5′ purine and employs the thermodynamic instability of the base pairing at the 5′ end of small RNA duplexes to recognize a 5′ adenine. On the other hand, *At*AGO6 possesses a strong 5′ adenine preference due to the C-terminal region of the PIWI domain (45). AGOs use a chaperone system for efficient duplex loading (4, 46, 47, 48), but little is known about the detailed molecular mechanism of preferentially selecting specific 5′-end nucleotides of guide RNAs. All RISC structures available to date show that the seed region of the loaded guide RNA is thoroughly anchored at its sugar-phosphate backbone, indicating that the 5′ nucleotide of guide RNA has to be discriminated by AGO at an early stage of duplex loading. Based on these data, I previously proposed a RISC assembly model in which the MID domain and PIWI-helical subdomain work together to recognize the preferred 5′ nucleotide before the AGO interacts with the sugar-phosphate backbone of the seed region (13, 35). This postulate is supported by the recent finding that plant AGO uses its PIWI-helical subdomain to establish a preference for 5′ adenine (45).

## Completion of the catalytic tetrad permits target cleavage

**Figure 3:**
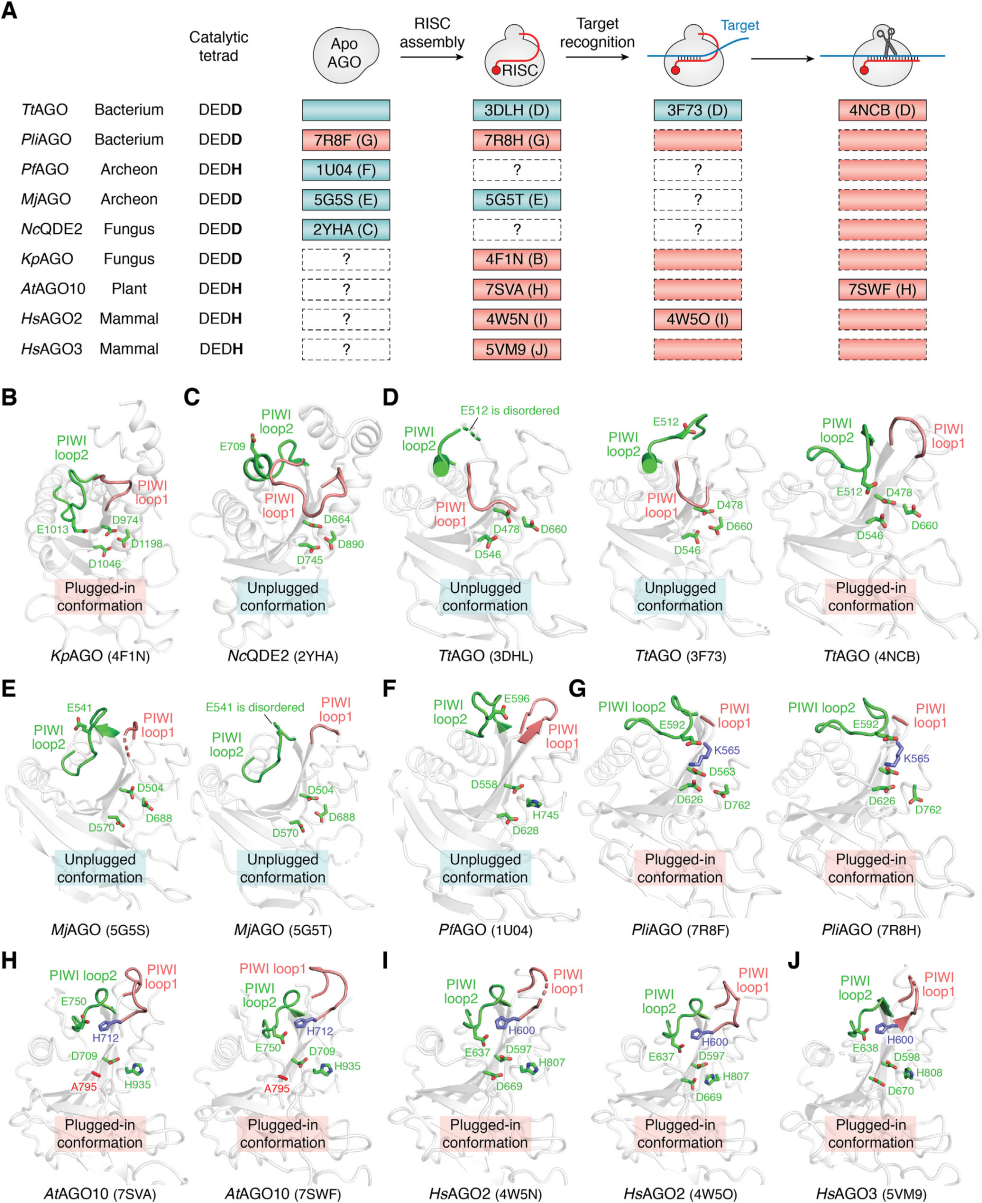
**Completion of the catalytic tetrad accompanied by different conformational changes.***A*, local structure around the catalytic tetrad in each state (*i.e.*, apo, RISC, seed-paired RISC, and extensively paired RISC). Plugged-in (*red*) and unplugged (*blue*) conformation confirmed by 3D structures are shown as *solid boxes* with the PDB ID of their representative structure. The alphabet B to J in *parenthesis* following the PDB ID indicates the panel of the corresponding structure in Figure 3, *B*–*J*. The *dotted white boxes* indicate states with uncertain conformations because no structure is available. States whose conformations can be deduced from the other known structures are *dotted red* (plugged-in) and *blue* (unplugged) *color boxes*. *B*–*J*, AGO structures from (*B*) *Kp*, *Kluyveromyces polysporus* (51), (*C*) *Nc*, *Neurospora crassa* (52), (*D*) *Tt*, *Thermus thermophilus* (53, 114, 115), (*E*) *Mj*, *Methanococcus jannaschii* (55), (*F*) *Pf*, *Pyrococcus furiosus* (49), (*G*) *Pli*, *Pseudooceanicola lipolyticus* (56), (*H*) *At*, *Arabidopsis thaliana* (33), and (*I* and *J*) *Hs*, *Homo sapiens* (7, 61). The positions of the PIWI loop1 (*pink*) and PIWI loop2 (*green*) and the catalytic tetrad (*green sticks*) are depicted for each AGO shown in (*A*). For clarification, only their PIWI domains are shown as a transparent *ribbon model*. The PDB ID of the structure is labeled at the *bottom*. Mutated residues are colored *red* in (*H*). AGO, Argonaute.

Structural studies of AGOs for the last decade have revealed different conformations in their apo form, RISC, seed pairing target complex, and extensively pairing target complex (Fig. 3*A*). The first crystal structures of prokaryotic AGOs visualized three residues arranged in their PIWI domain, similarly to the three out of the four catalytic residues of RNase H (49, 50). Since then, AGOs had been thought to employ the catalytic triad. However, the RISC structure of *Kluyveromyces polysporus* AGO (*Kp*AGO) showed E1013 poised over the previously identified catalytic triad, D974-D1046-D1198 (Fig. 3*B*) (51). Since the mutation of E1013 completely eliminated slicer activity, the residue was revealed as the fourth catalytic residue (51). On the other hand, the RNA-free structure of QDE2, an AGO ortholog of *Neurospora crassa* (*Nc*QDE2) (52), showed that E709, the glutamate residue corresponding to E1013 in *Kp*AGO, was located away from the catalytic triad (unplugged conformation) (Fig. 3, *A* and *C*) (51). The crystal structures of *Thermos thermophilus* AGO (*Tt*AGO) (53, 54) explained this difference in the glutamate arrangements of different AGOs. *Tt*AGO remains in the unplugged conformation even after the RISC binds to a target through its seed region (*i.e.*, *Tt*AGO–guide complex) (Fig. 3, *A* and *D* left and middle) but causes drastic changes in the local structures when the target is paired further with the guide beyond g15 to g16 (Fig. 3*A*) (51). As a result, the catalytic glutamate called the “glutamate finger” is rearranged to complete the catalytic DEDD tetrad (Fig. 3*D* Right) (51). Since the RNA-free and guide-bound crystal structures of *Methanocaldococcus jannaschii* AGO (*Mj*AGO) adopt an unplugged conformation (Fig. 3, *A* and *E*) (55), *Mj*AGO is expected to change the local structure during guide-target duplex propagation like *Tt*AGO. The only available structure of *Pyrococcus furiosus* AGO (*Pf*AGO) reflects the RNA-free state in an unplugged conformation (Fig. 3*F*) (49). It is not clear whether the conformational change of *Pf*AGO occurs during RISC assembly or target binding (Fig. 3*A*). These structural studies visualized the unplugged conformation of *Tt*AGO, *Pf*AGO, *Mj*AGO, and *Nc*QDE2, demonstrating that those AGOs must rearrange the glutamate finger to complete the catalytic tetrad for target cleavage.

In contrast, the structures of *Pseudooceanicola lipolyticus* AGO (*Pli*AGO) revealed that both the RNA-free form and RISC take a plugged-in conformation (Fig. 3, *A* and *G*) (56). However, the aliphatic side chain of K565 prevents the glutamate finger, E592, from reaching the rest of the catalytic tetrad (Fig. 3*G*). No target-bound structure has been available for *Pli*AGO. Given that the K565A mutation significantly reduced affinity for the target (56), the side chain of K565 must be involved in recognizing the sugar-phosphate backbone of the target strand, which probably makes space for E592 to complete the catalytic tetrad. Therefore, a different activation mechanism with this small local conformational change would render *Pli*AGO capable of forming the catalytic tetrad (54).

**Figure 4:**
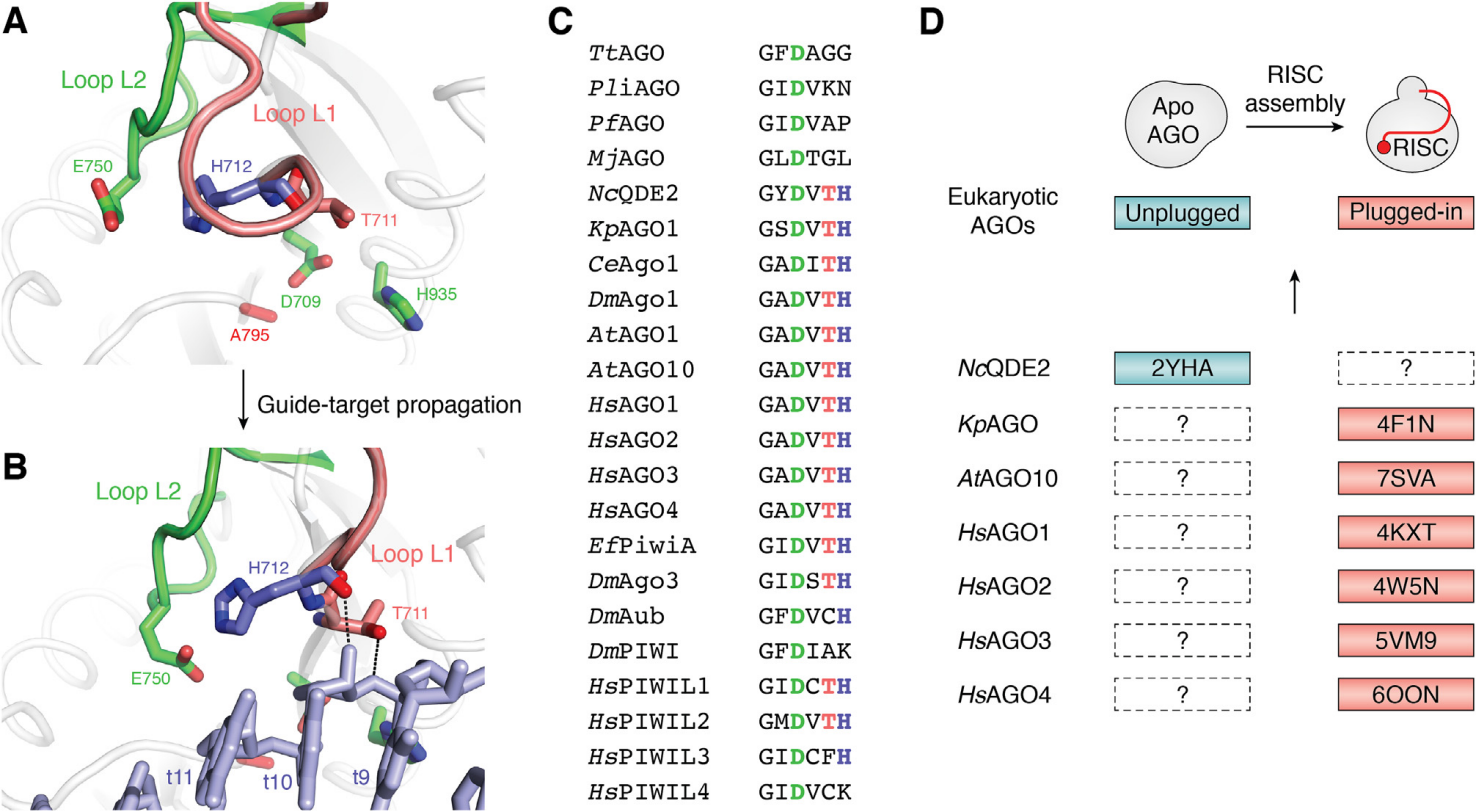
**Minimum structural changes to complete the catalytic tetrad in *At*AGO10.***A* and *B*, the movement of the glutamate finger, E750 (*green*), the conserved histidine, H712 (*blue*), and threonine, T711 (*pink*), upon extensive target pairing. Without RNA, E750 is located away from the other catalytic residues: D709, A795 (D795 in WT), and H935 (*A*). The main chain carbonyl of H712 and the side chain of T711 form hydrogen bonds with the 2′-OH and the phosphate group of t10 when the target is extensively paired with the guide (*B*). *C*, sequence alignment of selected AGOs and PIWIs. The aspartate, threonine, and histidine corresponding to D709 (*green*), T711 (*pink*), and H712 (*blue*) are conserved across eukaryotic AGOs and some PIWIs, but not in prokaryotes. *D*, model of the conformational change among eukaryotic AGOs. The previously determined structures of eukaryotic AGOs (*bottom*) reflect either an unplugged conformation in the apo form or the plugged-in conformation in the RISC. Neither a plugged-in conformation in the apo form nor an unplugged conformation in the RISC has been reported. The consistency may suggest that all eukaryotic AGOs remain an unplugged conformation without guide RNA and change to the plugged-in conformation upon RISC assembly (*top*). AGO, Argonaute; *At*AGO10, *Arabidopsis thaliana* AGO10; RISC, RNA-induced silencing complex.

The RISC structures of *At*AGO10, *Hs*AGO2, and *Hs*AGO3 show that their glutamate finger is already located close to the rest of the catalytic tetrad (Fig. 3*H* left, *I* left, and *J*) (33), indicating that like *Pli*AGO, these AGOs do not need a drastic local structure change to complete the catalytic tetrad during target recognition (Fig. 3*A*). The structure of the *At*AGO10-RISC shows that H712 is placed in the vicinity of the glutamate finger and, reminiscent of the K565 of *Pli*AGO, seems to limit accessibility of the catalytic triad to E750 (Fig. 3*G*). After the target is extensively paired with the guide (*i.e.*, g2-g16), the main chain carbonyl group of H712 and the side chain of T711 hydrogen-bond with the sugar-phosphate backbone of the target at t10 (Fig. 4, *A* and *B*). As a result, the glutamate finger, E750, can reach into the rest of the catalytic tetrad (Fig. 4*B*) (33). The corresponding threonine and histidine are conserved across eukaryotic AGOs, including *Kp*AGO, four *Hs*AGOs, and even *Nc*QDE2 (Fig. 4*C*). Meanwhile, structural studies indicate that apo *Nc*QDE2 remains in the unplugged conformation, while the RISCs of *Kp*AGO, *At*AGO10, and four *Hs*AGOs are in the plugged-in conformation (Fig. 4*D*) (7, 33, 35, 52, 57, 58, 59, 60). The most straightforward explanation for this discrepancy is that all eukaryotic AGOs convert the unplugged conformation to the plugged-in conformation during RISC assembly (Fig. 4*D*). All four *Hs*AGOs would have this conformational change, regardless of their slicer activity, because they conserved the histidine and threonine residues (Fig. 4*C*). In humans, the glutamate finger plays a critical role in target cleavage (7) but is dispensable for RISC assembly (35). In contrast, the threonine and histidine residues are less conserved among PIWI proteins and absent in prokaryotic AGOs (Fig. 4*C*). Almost all eukaryotic RISC structures show the glutamate finger poised over the remaining three catalytic residues (7, 35, 51, 57, 58, 60, 61), while the two RISC structures of *At*AGO10 (PDB ID: 7SAVA) and *Hs*AGO2 (PDB ID: 4F3T) show the glutamate finger is not proximal to the catalytic center (33, 59). The difference in these observations suggests that the glutamate finger is arranged for the catalytic reaction when the neighboring threonine and histidine residues anchor the t10, as seen in *At*AGO10 (Fig. 4*B*) (33).

Although prokaryotic AGOs could be readily purified as RNA-free form (49, 53, 55), there has been no success in purifying apo-form eukaryotic AGOs except for the MID-PIWI lobe of *Nc*QDE2 and the full-length *Hs*AGO2 (52, 59), due to their instability. Instead, the recombinant proteins of eukaryotic AGOs expressed in insect or *Escherichia coli* cells are known to incorporate the host cell’s endogenous small RNAs during the overproduction and form a stable RISC (7, 35, 51, 57, 58, 59, 60). It looks like eukaryotic AGOs lock their RISC structure with the plugged-in conformation and hinder the release of the loaded guide RNA.

## Metal ions

An early study revealed that when cleaving RNAs, *Drosophila melanogaster* AGO2 generates a hydroxyl group at the 3′ end of the 5′ cleavage product and a monophosphate group at the 5′ end of the 3′ cleavage product (62). This result ruled out the possibility that RISCs activate a 2′ hydroxyl group for a nucleophilic attack on the phosphodiester bond. Such a reaction, exemplified by RNase A, generates a 2′,3′-cyclic phosphate at the 3′ end of the 5′ cleavage product and a hydroxyl group at the 5′ end of the 3′ cleavage one. RNase A does not require a metal ion for its catalytic activity. Moreover, fly AGO2 cleaved RNAs in the presence of EGTA, which chelates Ca^2+^, but showed no slicing activity with EDTA, which chelates Mg^2+^, demonstrating that fly Ago2 requires magnesium ions for RNA cleavage (62). Similarly, Mg^2+^, Mn^2+^, Ni^2+^, and Co^2+^ catalytically activate *Hs*Ago2 better than Ca^2+^ (63). Since the nonbridging oxygen atom(s) of the phosphodiester bond between t10 and t11 are essential for RNA cleavage, one or both oxygen atoms were thought to be involved in forming the octahedral coordination of the magnesium ion (62).

**Figure 5:**
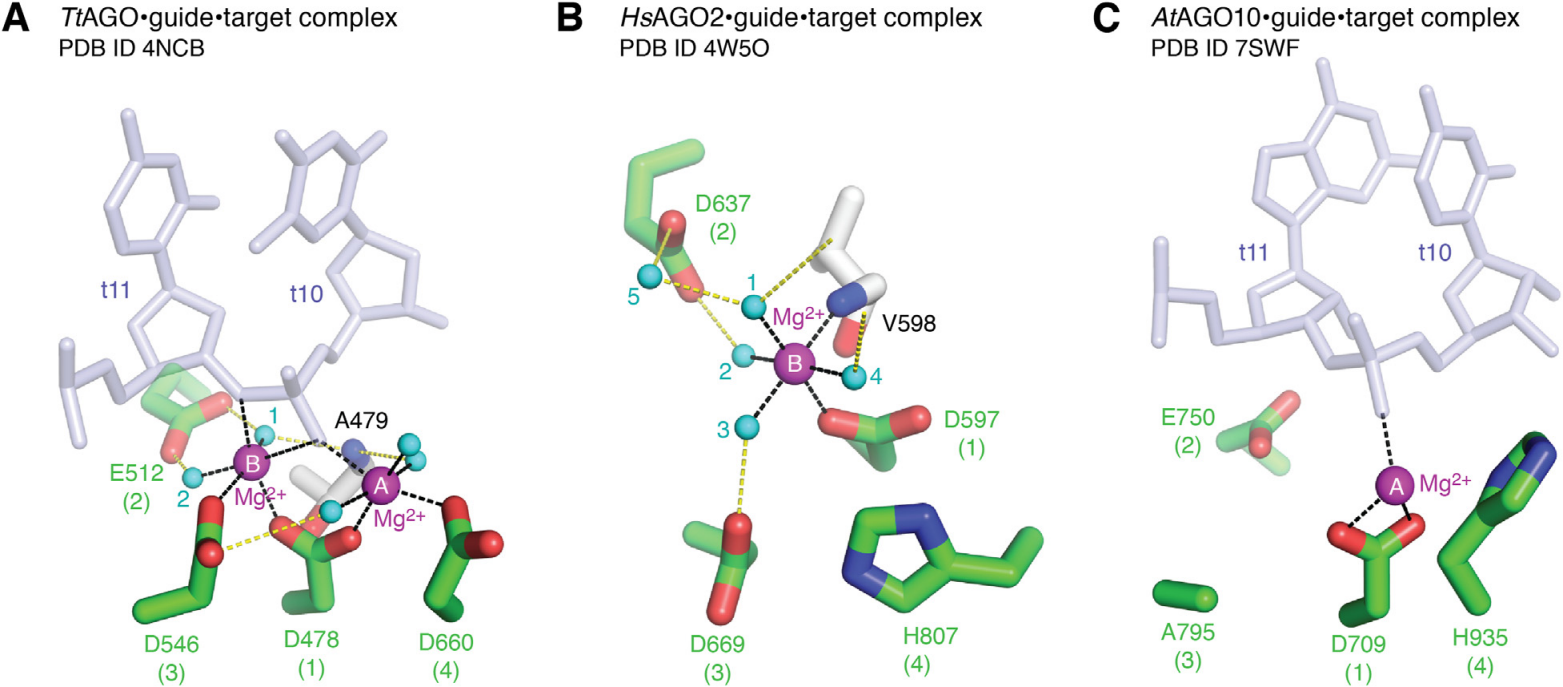
**Two metal ions at the catalytic tetrad.***A*, two Mg^2+^ ions (*magenta spheres*) at sites A and B chelate three out of the four catalytic residues (*green sticks*) and the water molecules (*cyan spheres*) in the crystal structure of the *Tt*AGO–guide–target complex (PDB ID: 4NCB). The arranged t10 and t11 are shown as *stick models* (*light blue*). Coordinate covalent bonds and hydrogen bonds are depicted as *black dotted lines* and *yellow dotted lines*, respectively. The number in the *parentheses* indicates the catalytic tetrad's first, second, third, or fourth residues. *B*, one Mg^2+^ ion at site B chelates the catalytic residue, D597, the amino nitrogen of V598, and four water molecules (*cyan spheres* 1–4) in the crystal structure of the *Hs*AGO2–guide–target complex (PDB ID: 4W5O). *C*, one Mg^2+^ ion at site A chelates the catalytic residue, D709, in the cryo-EM structure of the *At*AGO10–guide–target complex (PDB ID: 7SWF). The third catalytic residue is replaced with alanine (A795). *At*AGO10, *Arabidopsis thaliana* AGO10; *Tt*AGO, *Thermos thermophilus* AGO; HsAGO, human Argonaute.

**Figure 6:**
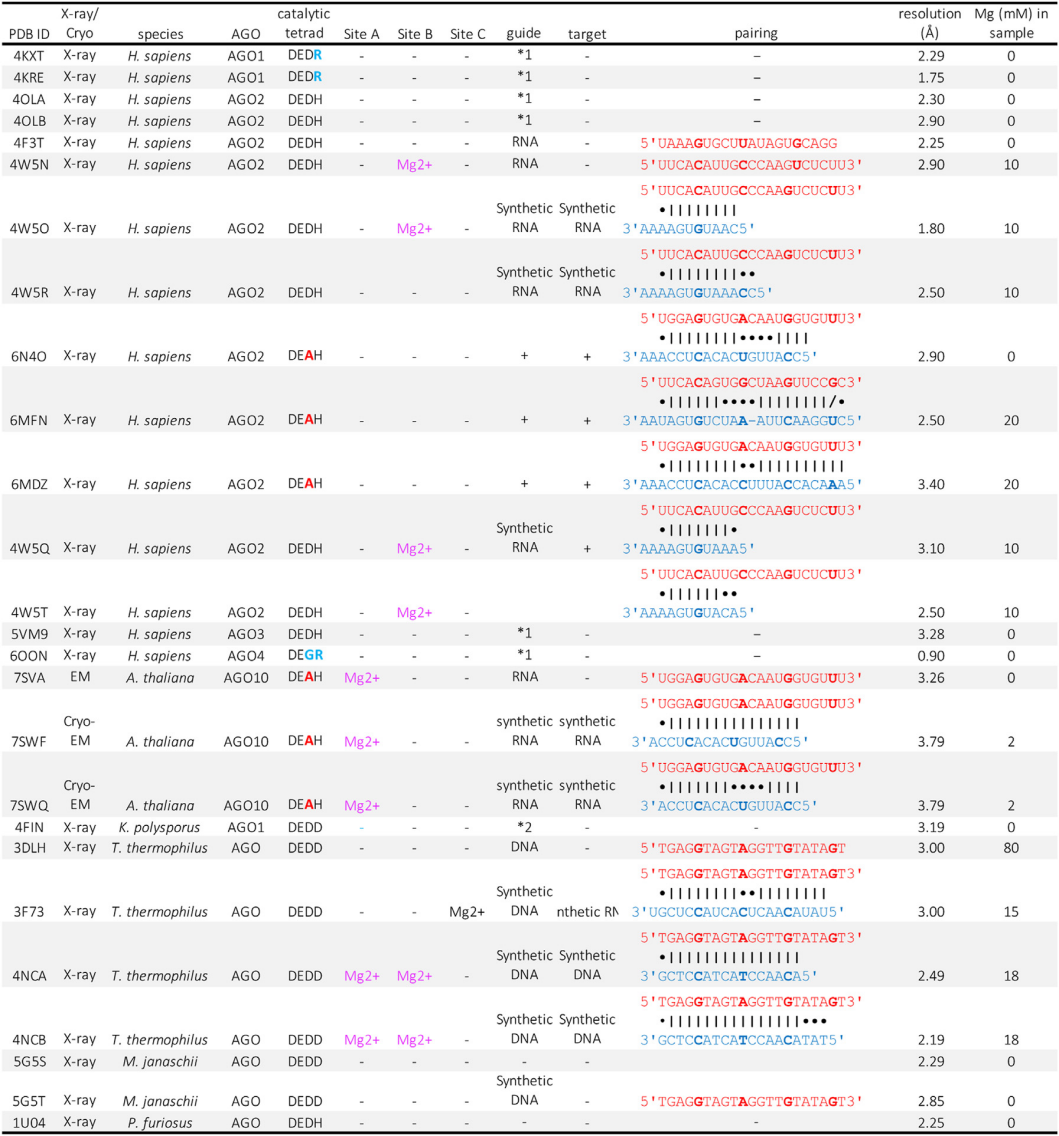
**AGO structures discussed in this review.***∗1*, copurified insect endogenous RNAs. *∗2*, copurified *Escherichia coli* endogenous RNAs. *∗3*, guide and target strands are colored *red* and *blue*, respectively. *∗4*, g5, g10, g15, g20, t5, t10, and t15 are *bold*. *∗5*, Watson–Crick base pairs are shown with "|," while G-U wobble pairs are depicted as "/.". *∗6*, mismatches are shown with "•". AGO, Argonaute.

Additional insight regarding the mechanism emerged from the crystal structure of the *Tt*AGO–guide–target complex that identified the two magnesium-binding sites (sites A and B in PDB ID: 4NCB) (Fig. 5*A*) (54). The catalytic reaction of *Tt*AGO was described in detail previously (see the previous review (15)). Briefly, one nonbridging oxygen of t10 coordinates with two Mg^2+^ ions at sites A and B, while the bridging oxygen of t11 (*i.e.*, 3′ O) coordinates with Mg^2+^ at site B (Fig. 5*A*). Comparison with bacterial ribonuclease H (RNase H) indicates that the Mg^2+^ at site B in *Tt*AGO is in the cleavage-compatible position (61). The glutamate finger, E512, hydrogen-bonds directly with the two water molecules that support the octahedral geometry at site B. *Tt*AGO has been the only AGO whose structure has two magnesium ions at sites A and B. Several *Hs*AGO2 structures showed the magnesium ion at only site B (Fig. 6). For example, the crystal structure of the *Hs*AGO2–guide–target complex (PDB ID: 4W5O) shows one octahedral coordination at site B where the carboxylate group of D597, the carbonyl group of V598, and four water molecules are chelated by the magnesium ion (Fig. 5,*B*). The glutamate finger, D637, hydrogen-bonds with two water molecules, one of which (water molecule 2 in Fig. 5*B*) coordinates with Mg^2+^, while the other (water molecule 5 in Fig. 5*B*) stabilizes the octahedral geometry indirectly (Fig. 5*B*). Based on the comparison with *Tt*AGO, the glutamate finger of *Hs*AGO2, E637, is expected to hydrogen-bond with the two water molecules chelated at site B (water molecules 1 and 2 in Fig. 5*B*) in the transition state (Fig. 5*A*).

*At*AGO10 and *Hs*AGO2 share the identical catalytic tetrad. To determine the cryo-EM structure of an *At*AGO10–guide–target complex, the third catalytic residue, D795, was replaced with alanine (Fig. 6) (33). The result provided the first eukaryotic RISC structure where the g2 to g16 of a 21-nt guide is extensively paired with a 16-nt complementary target (PDB ID: 7SWF). There was no metal ion at site B (Fig. 5*C*), indicating that the third catalytic residue is essential for metal binding to site B. Consistent with this, all structures of the *Hs*AGO2 D669A mutant, corresponding to *At*AGO10 D795A, have no metal ion at site B (Fig. 6). In contrast, the *At*AGO10 structure shows that site A is occupied by Mg^2+^ even with D795A (Fig. 5*C*), suggesting that metals can bind to site A independently of the third catalytic residue. The Mg^2+^ forms a coordinate covalent bond with the nonbridging oxygen of the phosphodiester bond between t10 and t11 (Fig. 5*C*), which is consistent with the Mg^2+^-dependent target recognition by *Tt*AGO (Fig. 5*A*). The fourth catalytic residue of *At*AGO10, H935, forms a coordinate covalent bond with the Mg^2+^ at site A when the guide is paired with the t2 to t8 and t13 to t16 but not the t9 to t12 (PDB ID: 7SWQ) (Fig. 6), suggesting the contribution of the fourth catalytic residue to the metal binding at site A. These observations could explain how *Hs*AGO4 lacks slicer activity, as it would be deficient in metal binding at both sites A and B because it has glycine and arginine at the third and fourth catalytic residue positions, respectively (Fig. 6) (35). The crystal structures of a *Hs*AGO1–guide complex show the altered local structure surrounding site B due to conserved segment 7 (cS7) (57, 60). Previous studies discussed the possibility that the cS7 prevents target RNAs from being arranged near the catalytic center (57, 60). This review raises the possible role of cS7 in disrupting the metal-binding site B. Thus, *Hs*AGO1 and *Hs*AGO4 slightly change their local structures and disturb one and two metal-binding sites, respectively, making them slicer deficient.

## Watson–Crick base pairing between g10 to t10 and g11 to t11 places two metal ions correctly for target cleavage

An extensive base pairing of the guide and target strands places the t10 and t11 in the vicinity of the conserved histidine (Fig. 4*B*). The resultant conformation creates two octahedral geometries with a high affinity for divalent metal ions underneath the scissile phosphate group. Thus, the RISC can coordinate the two metal ions only when an extensive base pairing ensures the perfect complementarity between the guide and target strands. Unlike other ribonucleases, such as RNase P, which use shape recognition in combination with specific nucleotide recognitions (64, 65, 66), RISCs can cleave highly structured RNAs without recognizing any tertiary structure of the target RNAs (67, 68). Therefore, placing the metal ions suitably only after an extensive guide-target pairing comprises a sophisticated system for AGOs working as guide-dependent endonucleases.

## A possible contribution of metal ions to target recognition

**Figure 7:**
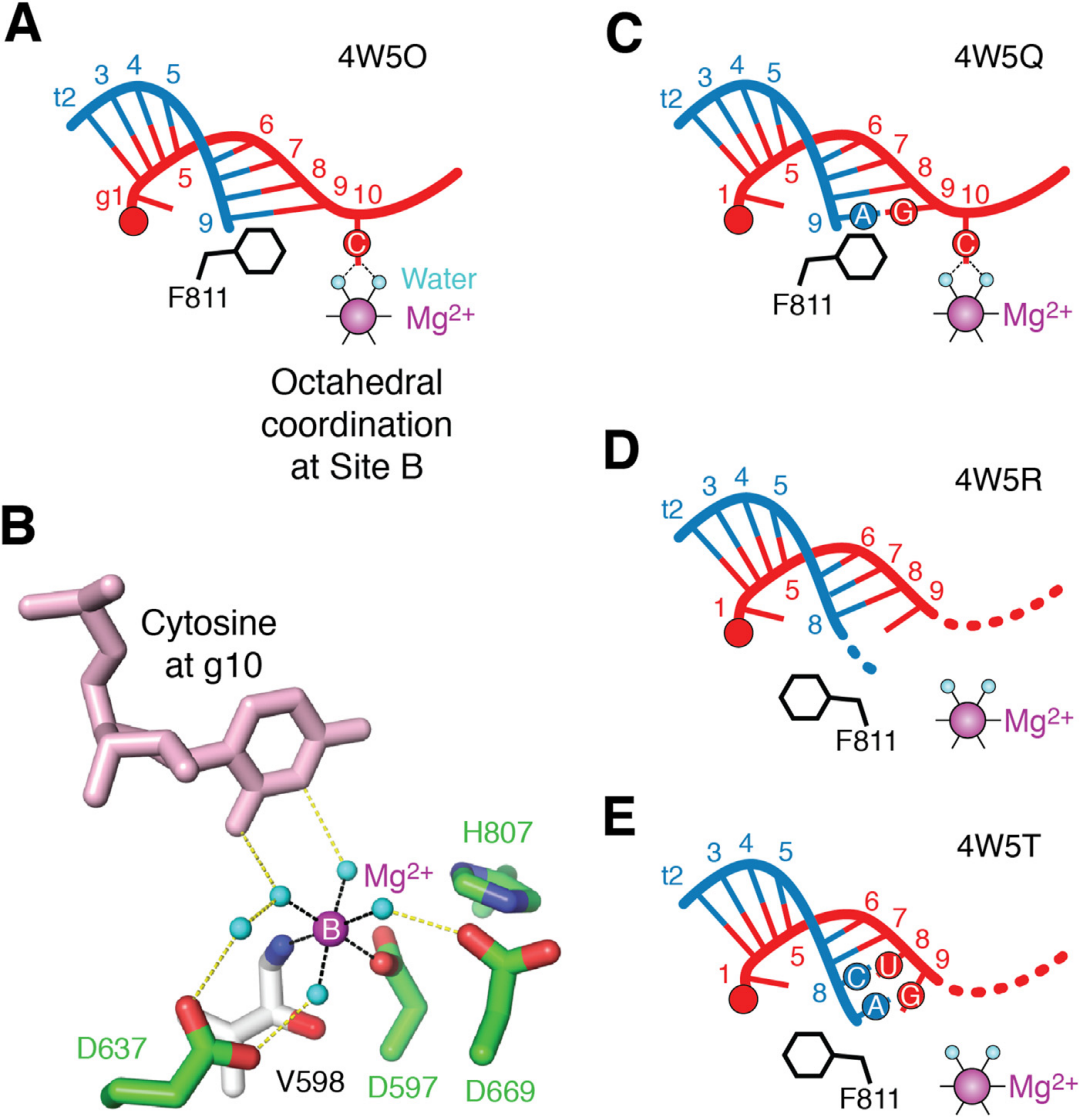
**A possible additional role for the catalytic metal ion in guide recognition.***A* and *C*, schematic of the cytidine at g10 anchored by the octahedral coordination at site B, as seen in PDB ID: 4W5O (*A*) and PDB ID: 4W5Q (*C*). The guide and target strands are colored *red* and *blue*, respectively. *B*, cytidine at g10 forms two hydrogen bonds with two water molecules (*cyan spheres*) chelated by Mg^2+^ (*magenta sphere*) at site B (PDB ID: 4W5O). The four catalytic residues (*green*) and the valine (*white*) are depicted as *stick models*. Coordinate covalent bonds and hydrogen bonds are shown as *black dotted lines* and *yellow dotted lines*, respectively. *D* and *E*, schematic of the unanchored cytidine at g10 when there is no base stacking with F811, as seen in PDB ID: 4W5R (*D*) and PDB ID: 4W5T (*E*).

In the *Hs*AGO2 structure (PDB ID: 4W5O), where a 21-nt guide is paired with the t2 to t9 of a 9-nt target, while the t9 is stacked with F811 (Figs. 6 and 7*A*), the cytidine at g10 hydrogen-bonds with two water molecules occupying the octahedral coordinates at site B (Fig. 7*B*). Cytosine is the only base capable of forming hydrogen bonds in this manner due to the carbonyl group at position 2 and the lone pair of the nitrogen at position 3, albeit the *syn* conformation of guanidine at g10 may form two hydrogen bonds, like a Hoogsteen base pair, with the octahedrally coordinated water molecules. The cytidine at g10 is arranged the same way in another *Hs*AGO2 structure (PDB ID: 4W5Q), where a 21-nt guide is paired with the t2 to t8 of a 9-nt target while the adenine at t9, forming a non-Watson–Crick base pair with the guanine at g9, is stacked with F811 (Figs. 6 and 7*C*) (61). In contrast, the cytidine at g10 is not anchored with the Mg^2+^ at site B in other *Hs*AGO2 structures (PDB IDs: 4W5R and 4W5T), whose base at t9 is not stacked with F811 (Figs. 6 and 7, *D* and *E*) (61).

The previously proposed target cleavage mechanism of *Hs*AGO2 indicates that after forming a duplex with the seed region (g2–g8), the target skips the central region (g9–g12) and resumes pairing with the 3′ supplementary region (g13–g16) (12, 36). The abovementioned four structures (PDB IDs: 4W5O, 4W5Q, 4W5R, and 4W5T) are thought to reflect the step, where *Hs*AGO2 recognizes a complementary seed and is about to proceed toward pairing with the 3′ supplementary region. The g10 occlusion at site B seen in the two *Hs*AGO2 structures (Fig. 7*B*) (PDB IDs: 4W5O and 4W5Q) may indicate a possible contribution of cytosine at g10 to preventing the continuous propagation of the guide-target duplex beyond g9. Mg^2+^ was detected only in the cocrystallized *Hs*AGO2 structures (Fig. 6), suggesting the low affinity of site B for Mg^2+^. The occluded cytidine at g10 may also contribute to retaining the metal ion and preorganizing the octahedral coordinate until the target is extensively paired with the 3′ supplementary region (g13–g16).

Since *Hs*AGO2 and *Hs*AGO3 have the same local structure around the catalytic center, *Hs*AGO3 may also use the Mg^2+^ at site B for target recognition. In contrast, this may not be the case for *Hs*AGO1 and *Hs*AGO4 because they alter the local structure surrounding site B. The difference may contribute to their unique target specificities.

## Enzymatic activation of AGO3 by shortening guide RNAs

The RNase III enzyme, Dicer, binds to long dsRNAs and hairpin-like precursor miRNAs (pre-miRNAs) to generate about 22-nt duplexes, which are loaded into AGOs to form RISCs (4). Therefore, mature miRNAs were defined as about 22 nt long (69). Meanwhile, previous studies, including RNA-seq analyses, found AGO-associated 19-nt or shorter RNAs, hereafter called tiny RNAs (tyRNAs) (70, 71). However, little was known about how tyRNAs were generated or whether they play different roles from miRNAs. We recently identified the biogenesis pathway of miRNA-derived tyRNAs, wherein interferon-stimulated gene 20 kda (ISG20) (72, 73, 74), three prime repair exonuclease 1 (75), and enhanced RNAi 1 (76), all of which are 3′→5′ exonucleases, trim *Hs*AGO-associated miRNAs to 13 to 14-nt tyRNAs (77). Notably, *Hs*AGO3-RISC became a slicer after ISG20 trimmed the loaded 23-nt miR-20a and 21-nt let-7a but not 22-nt miR-16 or 23-nt miR-19b. The former two tyRNAs capable of activating AGO3 were named cleavage-inducing tyRNAs (cityRNAs). *Hs*AGO3 showed much weaker slicer activity than *Hs*AGO2 when loaded with mature miRNAs (*i.e.*, ∼22 nt), although they share the same catalytic DEDH tetrad (7). The longstanding mystery of why *Hs*AGO3 has retained the catalytic tetrad throughout its molecular evolution was solved by discovering cityRNA-mediated activation of *Hs*AGO3 (8). This finding is also the first example that tyRNAs play a different role from their parental miRNAs.

The catalytic activation of *Hs*AGO3 by ISG20 may need to satisfy several prerequisites. First, the expression of ISG20 is regulated in normal conditions but drastically increases upon viral infection or stress (72, 73). Second, ISG20-mediated trimming of guide RNAs requires manganese, not magnesium (77). Third, while the cellular concentration of manganese is strictly controlled due to its toxicity in excess (78), the mitochondrion releases manganese into the cytoplasm in response to viral infection (79). Fourth, not all tyRNAs work as cityRNAs, indicating the dependency of *Hs*AGO3’s slicer activity on the length and sequence of guide RNAs (8). ISG20 was also identified as a human estrogen–regulated transcript, HEM45, and it has been thought to play a role in mediating estrogen control of cellular proliferation and differentiation (73). Since *Hs*AGO3 activation is strictly regulated, unlike *Hs*AGO2, the slicer activity may be harmful under normal conditions. Altogether, it is plausible that *Hs*AGO3 employs the slicer activity only in exceptional situations or cellular differentiation, which could have been beneficial during the evolution of animals. The target transcripts cleaved by *Hs*AGO3 remain to be studied.

## Targets cleaved by RISCs

Endogenous miRNAs' sequences are rarely perfectly complementary to their target RNAs. Cleavage of *HOXB8* mRNA by mouse AGO2 loaded with miR-196 was the first example of endogenous miRNAs, cleaving an extensively complementary sequence on mRNA (80). The following studies identified more miRNAs capable of cleaving mRNAs and noncoding RNAs, though some miRNAs have mismatched (es) with their target site (81, 82, 83, 84, 85, 86, 87, 88). A previous study using mouse embryonic stem cells revealed that most of the identified mRNA cleavages were executed by AGO2 or Drosha, while the rest of the cleavage sites remained to be studied further (84). At that time, vertebrate AGO2 was considered the only slicer among their four paralogs. The recent discovery of *Hs*AGO3 catalytic activation by cityRNAs (8) may explain some of the AGO2- and Drosha-independent mRNA cleavage.

Interestingly, cleavage-inducing miRNAs, such as miR-196 (80), miR-151-5p (81, 84, 88), miR-671-5p (86), and miR-98 (82), are conserved among vertebrates, in addition to their target RNAs. For example, *HOXB8* mRNA targeted by miR-196 has a critical function during the patterning of the vertebrate column (89). The cleavage sites of *N4BP1*, *ATPAF1*, and *LYPD3* mRNAs by miR-151-5p are conserved in mammals (81, 84). The cleavage site of a circular noncoding RNA, CDR1as, targeted by miR-671-5p is conserved in vertebrates (86), and miR-671-5p and *CDR1as* seem to have coevolved in placental mammals (90). It is also well known that pre-miRNAs themselves can become a substrate of vertebrate AGO2. For example, pre-miR-451 bypasses Dicer processing due to its short stem length and is directly loaded into AGO2 to undergo cleavage by the endonuclease activity (91, 92, 93). Subsequent trimming by poly(A)-specific ribonuclease, a 3′→5′ exonuclease, makes the AGO2-associated miR-451 about 21-nt in mature form (94). miR-451 is highly conserved in vertebrates (95) and the most abundant miRNA in erythrocytes to regulate maturation in mice (96) and zebrafish (97). Thus, erythropoiesis relies heavily on the expression of miR-451 (98, 99). The fact that all vertebrates have both primitive erythropoiesis (100) and miR-451 (93) demonstrates how AGO2 slicer activity was critical for the evolution of vertebrates.

## AGOs in conjunction with GW182

Compared to prokaryotic long AGOs, their eukaryotic counterparts expand their molecular size with various insertions (51). While the inside of bilobed RISC works as a target recognition and catalytic reaction platform, the exterior provides a scaffold to interact with many different proteins, such as GW182 proteins, upon the RISC assembly (35). Eukaryotic AGOs need GW182 proteins for efficient gene silencing (101, 102). For example, *Hs*AGOs have three tryptophan (Trp)-binding pockets that recognize GW182’s three AGO-binding sites, AGO-binding motifs I and II and AGO hook, each of which includes two tryptophan residues separated by about ten amino acid residues (35, 103, 104, 105). Two Trp-binding pockets on AGO must be mutated to drastically reduce the affinity for GW182 (103), indicating that AGOs use at least two Trp pockets to interact with GW182.

**Figure 8:**
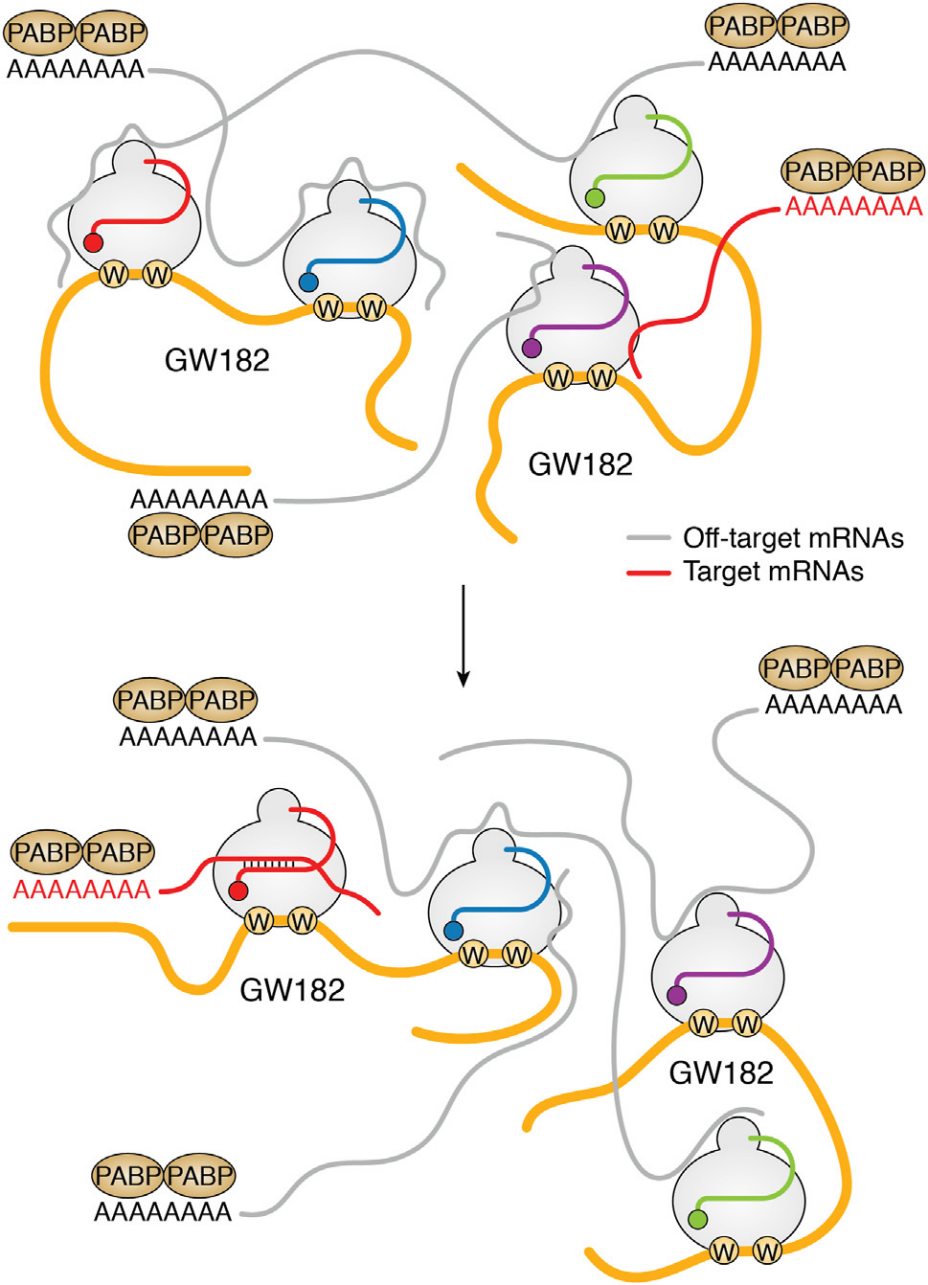
**The AGO-GW182-mRNA network accelerates target recognition.** GW182 proteins and off-target mRNAs are colored *yellow* and *gray*, respectively. *Top:* none of the RISCs finds their cognate target. But one target (*red*), which has a complementary sequence to the *red guide RNA*, interacts with a RISC loaded with a *purple guide*. *Bottom:* the transient association between AGOs and mRNAs due to sequence-independent interactions exchanges their partners, which enables one RISC to find the target mRNA. The rate of finding the target is accelerated when more mRNAs are recruited to each RISC. AGO, Argonaute; RISC, RNA-induced silencing complex.

Eukaryotic RISCs also have positively charged patches on their surface to recruit many mRNAs in a sequence-independent manner (13). To find specific target RNAs efficiently, eukaryotic AGOs must interact with GW182, forming a spider web-like network among them (Fig. 8) (13). The network would be reinforced by another interaction between the C-terminal domain of GW182 and polyadenylate-binding protein 1 (Fig. 8) (106). The three-way interaction would accelerate the rate at which a RISC finds the cognate target mRNAs (13). I believe the more off-target mRNAs each RISC brings; the more opportunities other RISCs have to find their proper targets. This mechanism is conceptually similar to “crowd-control” (107).

GW182 proteins are found in animals but not nonmetazoan organisms, such as unicellular organisms and plants (108). *Caenorhabditis elegans* possesses miRNA-specific AGOs, ALG-1 and AGL-2, which interact with the GW182 orthologs, AIN-1 and AIN-2, through their Trp-binding pockets (109). A Trp-binding pocket mutant of ALG-1 rescued the embryonic lethality due to the lack of *alg-1 and alg-2* in worms during larval stages, suggesting that GW182-free RISCs play critical roles during embryogenesis (109). Since prokaryotes do not have any GW182 homolog and most of their long AGOs retain nucleic acids-slicer activity, their AGOs likely work alone. Interestingly, GW182 is also missing in plants where miRNAs typically medicate RNA cleavage, although they also induce miRNA-mediated translational repression (110). In metazoans, miRNAs regulate gene expression mainly through translational repression or mRNA destabilization via deadenylation or decapping. The emergence of GW182 in an ancestor of metazoans might have enabled their AGOs to switch their gene regulation strategy from slicer-dependent to slicer-independent control.

## Perspectives

Eukaryotic AGOs have evolved from prokaryotic long AGOs, most of which retain a slicer activity to eradicate invading exogenous DNAs and RNAs (111, 112, 113). An ancestor of eukaryotes applied this defense system to regulate cellular gene expression. Due to the change, eukaryotes developed the infrastructure by encoding the genes of miRNAs in their genome, while incorporating the miRNA-binding sites in their mRNAs' 3′ UTR. In addition, it is sure that after the gene of the slicer-competent AGO was duplicated, one of the genes evolved as an AGO specialized for slicer-independent gene silencing. Using multiple miRNAs to regulate each mRNA seems beneficial in fine-tuning the mRNA concentration in a proper range to retain the homeostasis. As long as the target mRNAs remain intact and can resume the protein synthesis on demand, transient translational repression would be more energetically cost effective than target cleavage that irreversibly ruins the mRNAs. Destabilization and decapping of target mRNAs, which require many components (102), seem more energy-demanding than target cleavage. Why eukaryotes switched most of the gene silencing from the slicer-dependent manner to the slicer-independent one remains elusive. The slicer-independent silencing may have yet unidentified advantages for eukaryotes.

Thus, animals have switched almost all miRNA-mediated gene regulation to slicer-independent. Nevertheless, when more strict and drastic gene regulation is required, vertebrates still employ the AGO2 slicer activity during significant turning points, such as development, differentiation, and immune response. The evolution of life sometimes lets biology keep using an obsolete system. A typical example is ribosomes that retained the RNA-based catalytic system in transition from the “RNA world” to the “protein world.” The most plausible reason is that the ribosomal RNAs were already irreplaceable for protein synthesis. That is why life could not substitute any proteinaceous catalysts for the ribosomal RNAs, whereas proteins took over most of the other catalytic reactions. Similarly, after an ancestor of eukaryotes employed the defense system of prokaryotic long AGOs, the slicer-dependent target cleavage was superseded by the slicer-independent target regulation. However, the biological pressure must have maintained the use of the slicer activity for regulating some mRNAs, presumably because strict gene regulation was critical for developing vertebrates. Although miRNA-directed target cleavage has been interpreted based on the consensus that only AGO2 cleaved RNAs, a recent study revealed cityRNAs capable of catalytically activating *Hs*AGO3 (8, 77). This discovery prompts me to think that target cleavage by AGO2 and AGO3 in vertebrates is more broadly applicable than previously appreciated. Further studies are needed to investigate whether other vertebrates, animals, and plants have cityRNAs competent to convert AGO(s) to a slicer.

## Conflict of interest

The authors declare that they have no conflicts of interest with the contents of this article.
